# Heart rate and heart rate variability comparison between postural orthostatic tachycardia syndrome versus healthy participants; a systematic review and meta-analysis

**DOI:** 10.1186/s12872-019-01298-y

**Published:** 2019-12-30

**Authors:** Joel Swai, Zixuan Hu, Xiexiong Zhao, Tibera Rugambwa, Gui Ming

**Affiliations:** 1 Department of Internal medicine, Benjamin Mkapa Hospital, Dodoma city, Tanzania; 2 Department of Nephrology and Rheumatology, Xiangya Third Hospital, Central South University, Changsha city, Hunan People’s Republic of China; 3 Department of Gastroenterology, Xiangya Third Hospital, Central South University, Changsha city, Hunan People’s Republic of China; 4 Department of Cardiology, Xiangya Third Hospital, Central South University, Changsha city, Hunan People’s Republic of China; 5 Department of Obstetrics and Gynecology, Mbeya Zonal Referral Hospital, Mbeya city, Tanzania

**Keywords:** Postural orthostatic tachycardia syndrome, Heart rate, Heart rate variability, Head-up tilt test, Meta-analysis, Systematic review

## Abstract

**Background:**

A number of published literature has reported that, physiologically, heart rate variability (HRV) in patients with postural orthostatic tachycardia syndrome (POTS) to be greatly confounded by age, sex, race, physical fitness, and circadian rhythm. The purpose of this study was to compare between POTS patients versus healthy participants, in terms of heart rate (HR) and HRV after Head-Up tilt test (HUTT), by systematic review and meta-analysis of available published literature.

**Methods:**

MEDLINE (using PubMed interphase), EMBASE and SCOPUS were systematically searched for observational studies comparing POTS patients versus healthy patients, in terms of HR and HRV. HRV was grouped into Time and frequency domain outcome measurements. The time domain was measured as mean RR- interval and mean the square root of the mean of squares of successive R-R waves (rMSSD) in milliseconds. The frequency domain was measured as mean values of Low frequency power (LF), High frequency power (HF), LF/HF-ratio, LF-normalized units (LF(n.u)) and HF-normalized units (HF(n.u)). Demographic data, comorbidities, and mean values of HR, RR- interval, rMSSD, LF, HF, LF/HF-ratio, LF-(n.u) and H.F-n.u were extracted from each group and compared, by their mean differences as an overall outcome measure. Computer software, RevMan 5.3 was utilized, at a 95% significance level.

**Results:**

Twenty (20) eligible studies were found to report 717 POTS and 641 healthy participants. POTS group had a higher mean HR (*p* < 0.05), lower mean RR-Interval (p < 0.05), lower rMSSD (p < 0.05) than healthy participants. Furthermore, POTS group had lower mean HF(*p* > 0.05), lower mean LF(p > 0.05), and lower mean HF(n.u) (p > 0.05), higher LF/HF-Ratio (p > 0.05) and higher LF(n.u) (p > 0.05) as compared to healthy participants.

**Conclusion:**

POTS patients have a higher HR than healthy patients after HUTT and lower HRV in terms of time domain measure but not in terms of frequency domain measure. HR and time domain analyses of HRV are more reliable than frequency domain analysis in differentiating POTS patients from the healthy participants. We call upon sensitivity and specificity studies.

## Background

Blood circulation, blood pressure, and adequate tissue perfusion are closely coordinated with the autonomic nervous system in that, body postural changes will result in smaller and bearable changes in hemodynamics [1]. Inadequate blood volume, dysfunctional autonomic nervous system and sometimes, old-age and postprandial status, can result in altered hemodynamics when raising to the upright position (Orthostasis) [1, 2]. The altered hemodynamics results in a variety of symptoms collectively known as orthostatic intolerance (OI). Orthostatic intolerance could be classified as either Orthostatic Hypotension (OH), postprandial hypotension or Postural orthostatic tachycardia syndrome (POTS), also known as Chronic orthostatic intolerance [3].

Orthostatic intolerance presents with immediate clinical manifestations that follow cerebral hypoperfusion [4]. These could range from, generalized weakness, dizziness or lightheadedness, visual blurring or darkening of the visual fields, hypotension, tachycardia, pallor and in severe cases, syncope [4, 5]. Orthostatic hypotension is characterized by hypotension when raising to an upright position without a compensatory increase in heart rate (HR) while postprandial hypotension results into hypotension characterized by hypotension when raising to an upright position after eating. On the other hand, POTS is characterized by tachycardia and normal blood pressure [6].

POTS is the most prevalent form of orthostatic intolerance. It is diagnosed relying on a sustained HR increase of greater than 40 beats per minute or an increase to 120 beats per minute or greater within the first 10 min of tilt, without arterial hypotension. It is estimated that 3,000,000 Americans, suffer from this disorder at female: male ratio of 4:5.1 [7]. It occurs particularly in children and younger adults between 14 and 45 years, as compared to other OI which commonly occurs in the elderly [3]. Adverse manifestations such as hypotension and syncope almost never occur in POTS patients because they have preserved autonomic nervous functions [8].

Among others, the autonomic nervous function is one of the key players in maintaining hemodynamics and preventing POTS. Sympathetic denervation in lower extremities, preserved cardiac innervation and increased sympathetic activities (hyper-adrenergic state) have been shown to be sole etiologies of POTS [2, 3, 6, 8]. Other postulated theories include Cardiovascular deconditioning, abnormal venous function with reduced venous return, baroreflex abnormalities, hypovolemia and genetic abnormalities [4, 7]. To assess cardiac autonomic innervation and function, a number of tests have been developed with HRV widely used [9].

HRV analysis attempts to assess cardiac autonomic regulation through quantification of sinus rhythm variability. The sinus rhythm interval-time series is obtained from the QRS to QRS interval sequence of the electrocardiogram (ECG), by extracting only normal sinus to normal sinus in between two consecutive beats [9, 10]. High frequency alterations in sinus rhythm signify parasympathetic modulation, while slower variations reflect a combination of both parasympathetic and sympathetic modulation and non-autonomic factors. HRV measures are measured in two ways; time domain measures (TDM) and frequency domain measures (FDM) [9–11].

A few published literature have reported HRV to be greatly confounded by factors including age, sex, race and circadian rhythm. This study compared between POTS patients versus healthy patients, in terms of their HR and HRV after head-up tilt test (HUTT), by systematic review and meta-analysis of available published literature.

## Methods

### Eligibility criteria

This study included two kinds of participants; patients with POTS syndrome as cases, healthy participants as controls. The main outcomes were; HR and HRV as TDM and FDM. Only observational studies comparing suitable outcomes between the two groups were eligible for inclusion. To increase the external validity of this study, accessible literature from across the world was eligible for inclusion as long as they fulfill the aforementioned inclusion criteria. Only English published literature was eligible for inclusion.

### Information sources

Three online databases, namely PubMed, EMBASE and the SCOPUS were systematically searched to come up with eligible included studies. The searches were not be customized for searching within any restricted date ranges. Secondary referencing of eligible studies was done to extend the search scope and the last date of the search was 29th September 2019.

### The search

To generate a set of citations that are relevant to our study’s search question, an advanced search tool was used, utilizing MeSH terms and keywords in all of the three databases aforementioned. Using PubMed, MeSH terms were generated, a search was built and the advanced search was done as; (“Postural Orthostatic Tachycardia Syndrome”[Mesh]) AND “Heart Rate”[Mesh]. Again the search was repeated with; ((“postural orthostatic tachycardia syndrome”[MeSH Terms] OR (“postural”[All Fields] AND “orthostatic”[All Fields] AND “tachycardia”[All Fields] AND “syndrome”[All Fields]) OR “postural orthostatic tachycardia syndrome”[All Fields]) OR (“postural orthostatic tachycardia syndrome”[MeSH Terms] OR (“postural”[All Fields] AND “orthostatic”[All Fields] AND “tachycardia”[All Fields] AND “syndrome”[All Fields]) OR “postural orthostatic tachycardia syndrome”[All Fields] OR “pots”[All Fields])) AND ((“heart rate”[MeSH Terms] OR (“heart”[All Fields] AND “rate”[All Fields]) OR “heart rate”[All Fields]) AND variability [All Fields]). Using EMASE, on the other hand, advanced search tool was utilized firstly using MeSH terms ((postural AND orthostatic AND tachycardia AND syndrome OR pots) AND heart AND rate) and then a repeated by using a combination of key words (postural AND orthostatic AND tachycardia AND syndrome OR pots) AND heart AND rate AND variability. The searches were independently performed by two authors; JS and XZ. Results were exported to computer software, *EndNote X9 (Builld 12,062)* which was used to manage and keep track of references throughout this study.

### Study selection process

**Fig. 1:**
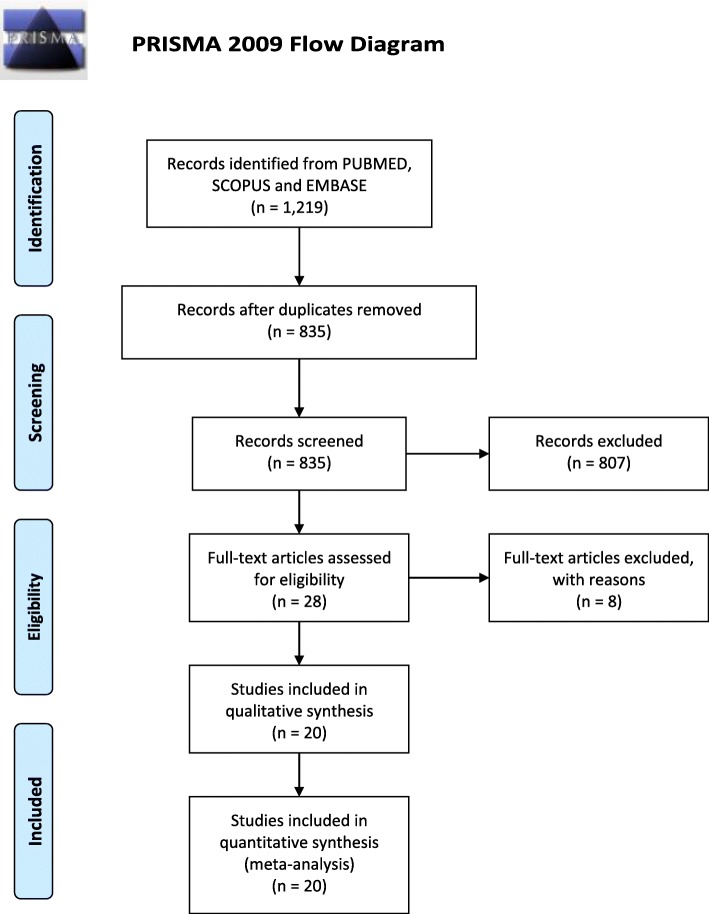
Study search, screening and selection process. PRISMA 2009 flow diagram illustrating study search, screening and selection process

All studies resulting from online database search independently conducted by two authors were initially screened by their titles and abstracts to initially assess their relevance to our study question. This was level-one screening and was done independently by two authors, JS and XZ. Compiled results of level-one screening were exported to EndNote software and then searched for their full-text articles. Level-two screening involved assessing the retrieved full text articles for eligibility for inclusion or exclusion. Any differences of thoughts in the search process were settled by the third author, TR. The entire study search, screening, and selection are summarized in Fig. 1**.**

### Data extraction

Before the data extraction process from full-text articles meting eligibility criteria for inclusion, assessment for methodological biases was done. PRISMA (preferred reporting items for systematic reviews and meta-analyses) tool [12] was used for this study write-up to minimize reporting bias.

The process of data extraction was independently performed by two authors, namely; JS and XZ. Any difference in thoughts was settled by the third author, TR. Data collected included participants’ demographics, study characteristics and reported outcomes in line with our study question.

Demographic data included participants’ mean/median ages, setting and sample sizes in each group. HUTT procedure details; angle of tilt, time of tilt, duration of orthostasis and device used to measure HR and HRV; whether ECG or Holter. Diagnoses and comorbidities among participants were also recorded.

In line with this study question, two outcomes were recorded from the eligible studies; HR and HRV measured by either TDM and FDM. These outcomes were recorded in both comparison groups.

### Analysis

Data were analyzed separately according to the two main outcomes of interest. TDM was sub-grouped into RR interval and rMSSD while the FDM outcome was sub-grouped into LF, HF, LF/HF-ratio, LF (n.u) and HF (n.u). In that case, comparison of TDM between POTS versus healthy participants groups was in terms of the mean differences of RR-Interval and rMSSD. On the other hand, comparison of FDM between POTS versus healthy participants groups was in terms of the mean differences of LF, HF, LF/HF-ratio, LF (n.u) and HF (n.u).

The overall effect of POTS was diagrammatically be depicted by forest-plots. Data synthesis, analysis, and generation of forest-plots were done utilizing computer software, ***Review Manager (RevMan Version 5.3)***. The software was customized to a random or fixed effect model depending on the heterogeneity (I^2^) of the studies when analyzing the outcomes. The fixed effect model was used when I^2^ was less than 50% and the random effect model was used when I^2^ was more than 50%.

### Assumptions and simplifications

For this study purpose, all participants were considered to have been correctly diagnosed and correctly classified as to be having POTS, or otherwise healthy. All participants, despite the study country, were considered to have received standard care.

## Results

### Study selection

The literature search identified a total of twenty-eight [13] studies that seemed relevant and were sought for full-text. Eight of these were excluded due to various reasons; *Nakao* et al. (2012) [14] used comorbid than healthy control; *Goldstein* et al. (2005) [15] did not assess our outcome of interest; *Yoshiuchi* et al. (2004) [16] used POTS participants comorbid with chronic fatigue syndrome; Singer et al. (2003) [17] intervened the control group with isoproterenol infusion similarly *Freitas* et al. (2000) [18] who intervened with cardio-selective beta-blocker and/or fludrocortisone and *Stewart* et al. (2007) [19] who employed hand-grip maneuver than HUTT. Furthermore, *Bongiovanni* et al. (2013) [20], and *Aoki* et al. (2008) [21] were excluded for not accessible full-text and use of Japanese language in the full-text retrieved, respectively. A total of twenty [21] studies fulfilled the eligible criteria for inclusion. Figure 1**,** summarizes search results, screening, and selection process.

### Study characteristics

Table 1 summarizes the study characteristics of our twenty [21] studies that were eligible for inclusion in our study. A total number of participants reported was 1358, of these, 717 POTS and 641 were healthy participants. Regarding participants demographics, while other studies recruited both gender equally [38], other only recruited one gender participants [29], and other studies randomly involved both gender [23]. While other studies matched the groups by age [15, 29], other studies did not [25]. Furthermore, the majority of studies reported participants’ ages central tendencies by mean, two studies utilized median instead [28, 40]. While other studies used a larger sample size [39], other used smaller sample sizes [13].

**Table 1:** Study characteristics

Study, Year	Study size (POTS, Healthy)	Mean Aged (POTS, Healthy)	Matched case-control or not?	Duration of HR/HRV parameter measurement (Angle of tilt)	Orthostasis induction method	Country of study	Outcome Recorded
Jacob 2019 [22]	12,10	30 ± 1.8, 32 ± 3	Unmatched	30 Minutes (75^0^)	HUTT	Israel	**HF, HR**
Owens 2018 [23]	20,20	36 ± 10.84, 35 ± 7.56	Unmatched	10 Minutes (60^0^)	HUTT	UK	**HF, LF**
Goff 2017 [24]	9,20	NA, NA	Unmatched	24 Hours	Daily life activity	Australia	**rMSSD**
Moon 2016 [25]	46,67	28.9 ± 1.9, 49.4 ± 2.1	Unmatched	20 Minutes	Active standing	Korea Republic	**HR**
Freitas 2015 [26]	10,12	29.4 ± 8.5, 33.8 ± 5.9	Matched	40 min (70^0^)	HUTT	Portugal	**HR, HF**
Yoshida 2014 [27]	70,38	13.7 ± 0.1, 13.5 ± 0.1	Unmatched	7 min (90^0^)	Active standing	Japan	**HR, LF/HF-Ratio**
Medow 2014 [28]	12,19	Median: 20.8, 21.4	Unmatched	10 Minutes (70^0^)	HUTT	USA	**HR, LF(n.u), LF(n.u)**
Mallien 2014 [29]	38,31	25.3 ± 7, 26.2 ± 6.3	Matched	Overnight	HUTT	Germany	**HR, LF, HF, LF/HF-Ratio**
Plash 2013 [30]	15,15	36 ± 3, 33 ± 2	Unmatched	30 Minutes	Active standing	USA	**HR**
Ocon 2012 [31]	16,20	21 ± 1, 23 ± 1		10 Minutes (75^0^)	HUTT	USA	**HR**
Brewster 2012 [32]	54,26	35 ± 2, 27 ± 1	Unmatched	5 Minutes	Active standing	USA	**HR**
Galbreath 2011 [33]	17,17	27 ± 9, 31 ± 10	Unmatched	5 Minutes (60^0^)	HUTT	USA	**HR, HF, LF, rMSSD, RR-Interval, HF (n.u), LF (n.u)**
Baumert 2011 [34]	13,12	(32 ± 13, 23 ± 2),	Unmatched	10 Minutes (40^0^)	HUTT	Australia	**HR**
Fu 2010 [35]	27,16	26 (21–33), 28 (23,35)	Unmatched	45 Minutes (60%) *grip	HUTT	USA	**HR**
Ocon 2009 [13]	9,7	NA, NA	Unmatched	10 Minutes (70^0^)	HUTT	USA	**RR-Interval, LF, HF, LF/HF-Ratio, LF(n.u), HF(n.u), HR**
Garland 2007 [36]	150,63	34.5 ± 10.7, 30.2 ± 9.3	Unmatched	5 Minutes	Active standing	USA	**HR**
Stewart 2006 [37]	20,10	17 ± 2, 17 ± 1	Matched	10 Minutes (70^0^)	HUTT	USA	**HR, LF/HR-Ratio, HF, LF**
Meier 2006 [38]	21,39	15.5 ± 2.2, 11.7 ± 2.7	Unmatched	12 Minutes (60^0^)	HUTT	The Netherlands	**HR**
Garland 2005 [39]	136,191	29.1 ± 8.0, 32.2 ± 9.9	Unmatched	30 Minutes (60^0^)	HUTT	USA	**HR**
Stewart 2000 [40]	22,10	Median: 15.2, 15.8	Unmatched	30 Minutes (70^0^)	HUTT	USA	**HR, HF(n.u), LF(n.u), LF/HF-Ratio, HF, LF, rMSSD, RR-Interval**

*POTS* Postural orthostatic tachycardia, *rMSSD* square root of mean of squares of successive R-R interval, *LF* Low frequency power, *HF* High frequency power, *LF(n.u)* Low frequency power -normalized units, *HF(n.u)* High frequency power -normalized units, *HR* Heart Rate, *NA* Data not accessed

All twenty studies were case-control observational studies and none was interventional. These were conducted in different settings from a diverse number of countries all around the world. Eleven studies were done in the USA, two in Australia and other were conducted in Israel [22], UK [23], Portugal [26], Japan [27], Germany [29], Korea republic [25] and The Netherlands [38], each contributing one study. This was thought to increase the external validity of this study.

Despite the fact that the search was not confined to any specified range of dates, none of the included studies was found to have been published before the year 2000. Fifteen studies (75%) were published in the last decade.

Different studies reported different outcomes, but all aligned with our study questions. Eighteen studies compared HR, three studies compared RR-Interval, three studies compared to rMSSD, six studies compared LF, eight studies compared HF, six compared LF/HF-Ratio and four studies compared LF(n.u) and HF(n.u) each. Studies comparing similar outcomes were analyzed together in the same forest-plot.

### Sources of bias

All 20 eligible articles included in this study were assessed for risk of bias in two levels; at study level and at the review level. Regarding study level bias assessment; different studies involved a different numbers of sample sizes. Other studies included a large number of participants [39] while other used low [13]. It follows that large sample sizes are more representative of the general population as compared to small sample sized studies. Furthermore, none of these 20 studies reported having had calculated the required sample size prior to their conduction.

Despite the fact that all studies were similar in that, they compared POTS versus healthy participants, some studies matched the comparison groups to reduce confounding factors while other studies did not [24]. This might have introduced confounding factors to our study as factors such as female gender, BMI, physical fitness and race, each has been reported to independently alter HRV [41].

Different studies utilized different methods to induce orthostasis, with others using the HUTT and others applying active standing [27]. Whether HUTT or active standing was used to induce orthostasis, different durations, ranging from 5 to 45 min, were applied depending on the participant’s tolerance to orthostasis. Furthermore, different angles of tilt were set, ranging from 40^0^ [34] to 75^0^ in other studies. While the majority involved awake patients, other studies [29] utilized sleeping participants. While other studies used ECG to measure HRV in a short session [35], others used the Holter device to record mean HRV per 24 h while participants are carrying on with their daily activities [24]. These different conditions were thought to increase heterogeneity hence influence our results.

At the review level, on the other hand, a number of loopholes for biases were also identified. Although, other studies had our data of interest, readily available to extract from tables in text, from one [23] study data had to be extracted by estimations and extrapolation from a graphical figure. This led to conducting sensitivity analysis excluding this study. Furthermore, the overall mean ages of POTS and/or healthy group could not be calculated because data could not be accessed in other studies [13, 24], because the median was utilized than the mean [28, 40].

### Heart rate (HR)

**Fig. 2:**
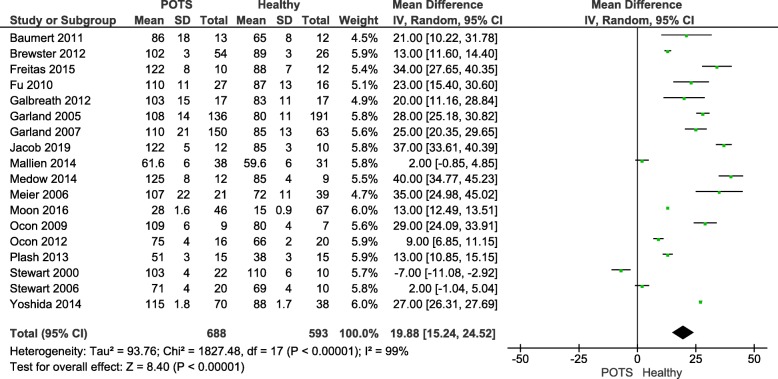
Heart rate comparison between postural orthostatic tachycardia syndrome versus healthy participants**.** A forest plot illustrating eighteen of twenty eligible studies that compared the mean heart rate outcome between postural orthostatic tachycardia syndrome versus healthy participants

Figure 2 illustrates eighteen of twenty eligible studies that compared HR outcomes between POTS versus Healthy participants. The overall mean difference between the two groups was 19.88 (15.24, 24.52) signifying a higher HR in the POTS group. This difference reached statistical significance (*P*-value< 0.05). A random-effect model was used since heterogeneity, I^2^, was 99% (i.e. I^2^ > 50%).

### RR- interval

**Fig. 3:**
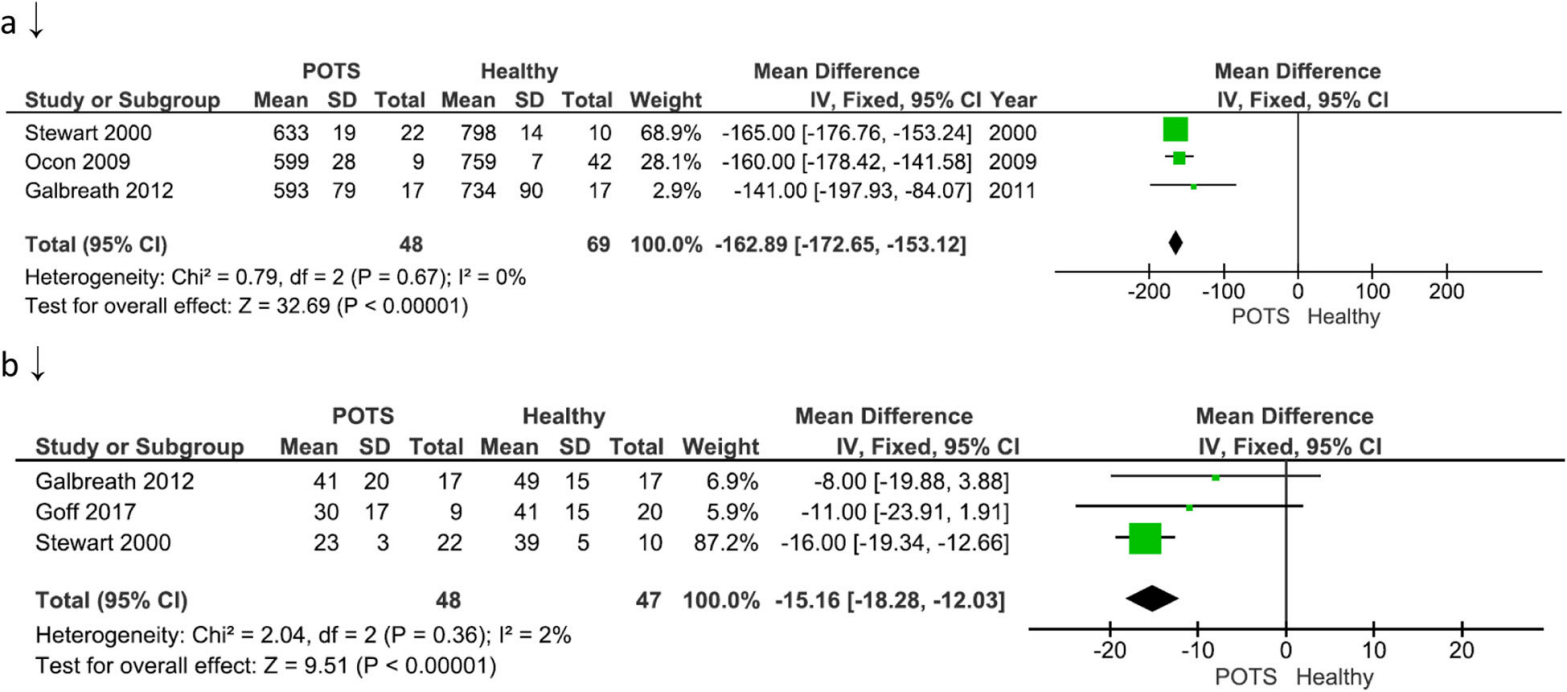
Time domain measure comparison between postural orthostatic tachycardia syndrome versus healthy participants. **a** illustrates three of twenty eligible studies that compared time domain measure outcome in terms of mean RR-intervals; **b** illustrates three of twenty eligible studies that compared time domain measure outcome in terms of mean rMSSD

Figure 3a illustrates three of twenty eligible studies that compared TDM outcomes between POTS versus Healthy participants in terms of mean RR intervals. The overall mean difference between the two groups was − 162.89 (− 172.65, − 153.12) signifying lower HRV in terms of RR-interval in the POTS group. This difference reached statistical significance (*P*-value< 0.05). Fixed-effect model was used since heterogeneity, I^2^, was 0% (i.e. I^2^ < 50%).

### The root of the mean of squares of successive R-R interval differences (rMSSD)

Figure 3b illustrates three of twenty eligible studies that compared TDM outcomes between POTS versus Healthy participants in terms of rMSSD. The overall mean difference between the two groups was − 15.16 (− 18.28, − 12.03) signifying lower HRV in terms of rMSSD in the POTS group. The difference reached statistical significance (*P*-value< 0.05). A fixed-effect model was used since heterogeneity, I^2^, was 2% (i.e. I^2^ < 50%).

### Low frequency power (LF)

**Fig. 4:**
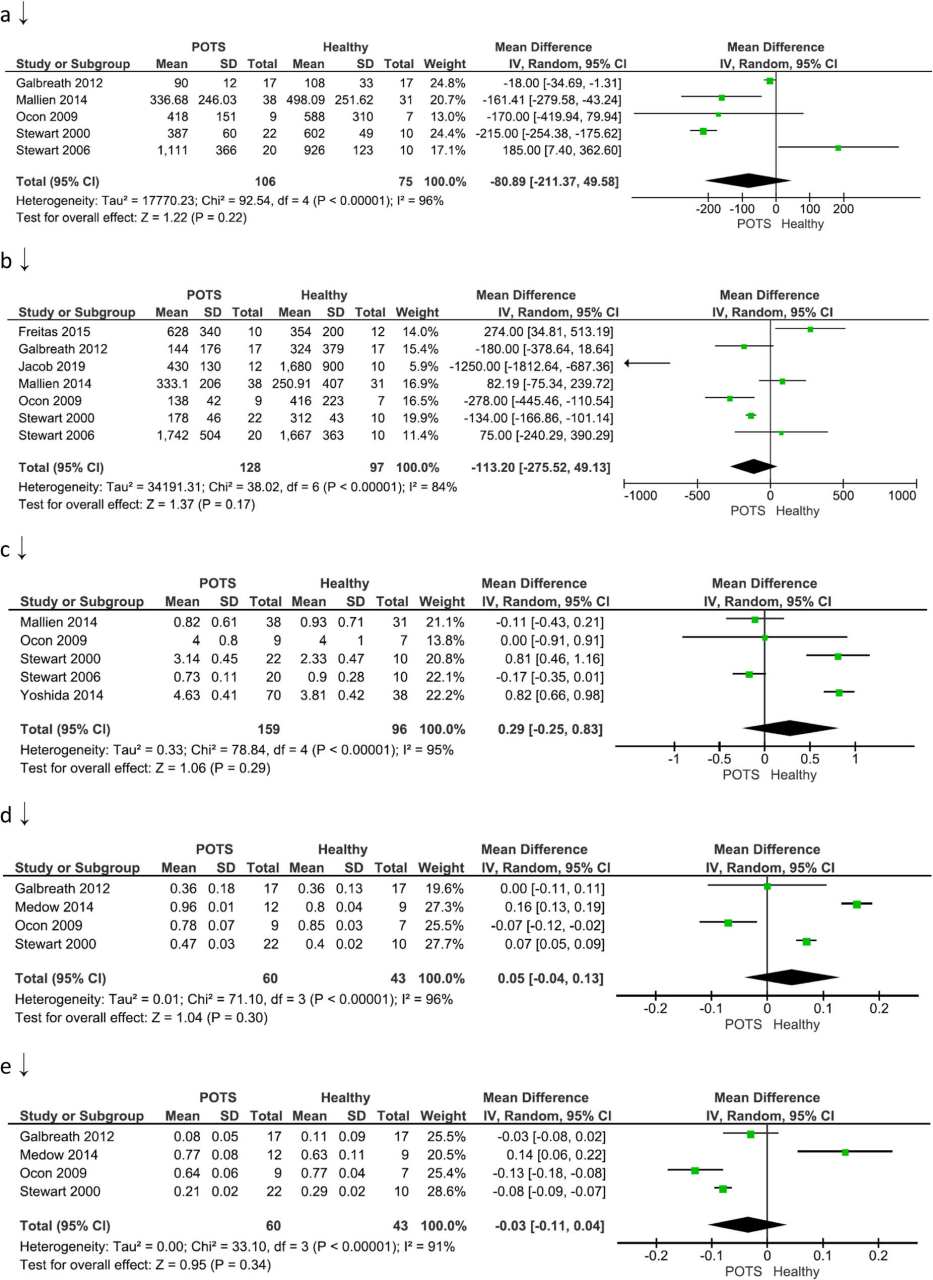
Frequency domain measure between postural orthostatic tachycardia syndrome versus healthy participants. **a** illustrates five of twenty eligible studies that compared the frequency domain measure outcome in terms of mean LF. **b** illustrates seven of twenty eligible studies that compared the frequency domain measure outcome in terms of mean HF. **c** illustrates five of twenty eligible studies that compared frequency domain measure in terms of mean LF/HF- ratio; **d** illustrates four of twenty eligible studies that compared the frequency domain measure outcome in terms of mean LF (n.u); **e** illustrates four of twenty eligible studies that compared the frequency domain measure outcome in terms of mean HF (n.u)

Figure 4a illustrates five of twenty eligible studies that compared the FDM outcomes between POTS versus Healthy participants in terms of LF. The overall mean difference between the two groups was − 80.89 (− 211.37, 49.58) milliseconds^2^ signifying lower HRV in terms of LF in the POTS group. The difference, however, did not reach statistical significance (*P*-value> 0.05). A random-effect model was used since heterogeneity, I^2^, was 96% (i.e. I^2^ > 50%).

### High frequency power (HF)

Figure 4b illustrates seven of twenty eligible studies that compared the FDM outcome between POTS versus Healthy participants in terms of HF. The overall mean difference between the two groups was − 113.20 (− 275.52, 49.13) milliseconds^2^ signifying lower HRV in terms of HF in the POTS group. The difference did not reach statistical significance (*P*-value> 0.05). A random-effect model was used since heterogeneity, I^2^, was 84% (i.e. I^2^ > 50%).

### Low frequency power /high frequency power ratio (LF/HF- ratio)

Figure 4c illustrates five of twenty eligible studies that compared the FDM outcome between POTS versus Healthy participants in terms of the LF/HF- ratio. The overall mean difference between the two groups was 0.29 (− 0.25, 0.83) signifying higher HRV in terms of the LF/HF- ratio in the POTS group. The difference did not reach statistical significance (*P*-value> 0.05). A random-effect model was used since heterogeneity, I^2^, was 95% (i.e. I^2^ > 50%).

### Low frequency power-normalized unit

Figure 4d illustrates four of twenty eligible studies that compared the FDM outcome between POTS versus Healthy participants in terms of LF (n.u). The overall mean difference between the two groups was 0.05 (− 0.04, 0.13) signifying higher HRV in terms of LF (n.u.) in the POTS group. The difference, however, did not reach statistical significance (*P*-value> 0.05). A random-effect model was used since heterogeneity, I^2^, was 96% (i.e. I^2^ > 50%).

### High frequency power-normalized unit

Figure 4e illustrates four of twenty eligible studies that compared the FDM outcome between POTS versus Healthy participants in terms of HF (n.u). The overall mean difference between the two groups was − 0.03 (− 0.11, 0.04) signifying lower Heart variability in terms of HF (n.u.) in the POTS group. The difference, however, did not reach statistical significance (*P*-value> 0.05). A random-effect model was used since heterogeneity, I^2^, was 91% (i.e. I^2^ > 50%).

### Sensitivity analysis

Eliminating three studies; one study for utilizing 24 h parameters recording [24]; another study for including a period of parameters measurements during sleeping [29]; and one study [23], in which data were collected by estimates and extrapolations from a graphical figure, none of the outcome results changed statistical significance. The newly, obtained results were as follows; mean difference rMSSD = − 15.41(− 18.63,-12.2), *p*-Value< 0.00001, I^2^ = 38%; mean difference HF = -156(− 344.05,31.17), *p*-Value = 0.1, I^2=^84%; mean difference LF/HF =0.39(− 0.24,1.03), *p*-Value = 0.23, I^2^ = 96%; and lastly, mean HR =20.98(16.27,25.69), *p*-Value< 0.00001, I^2^ = 99%.

### Publication bias

**Fig. 5:**
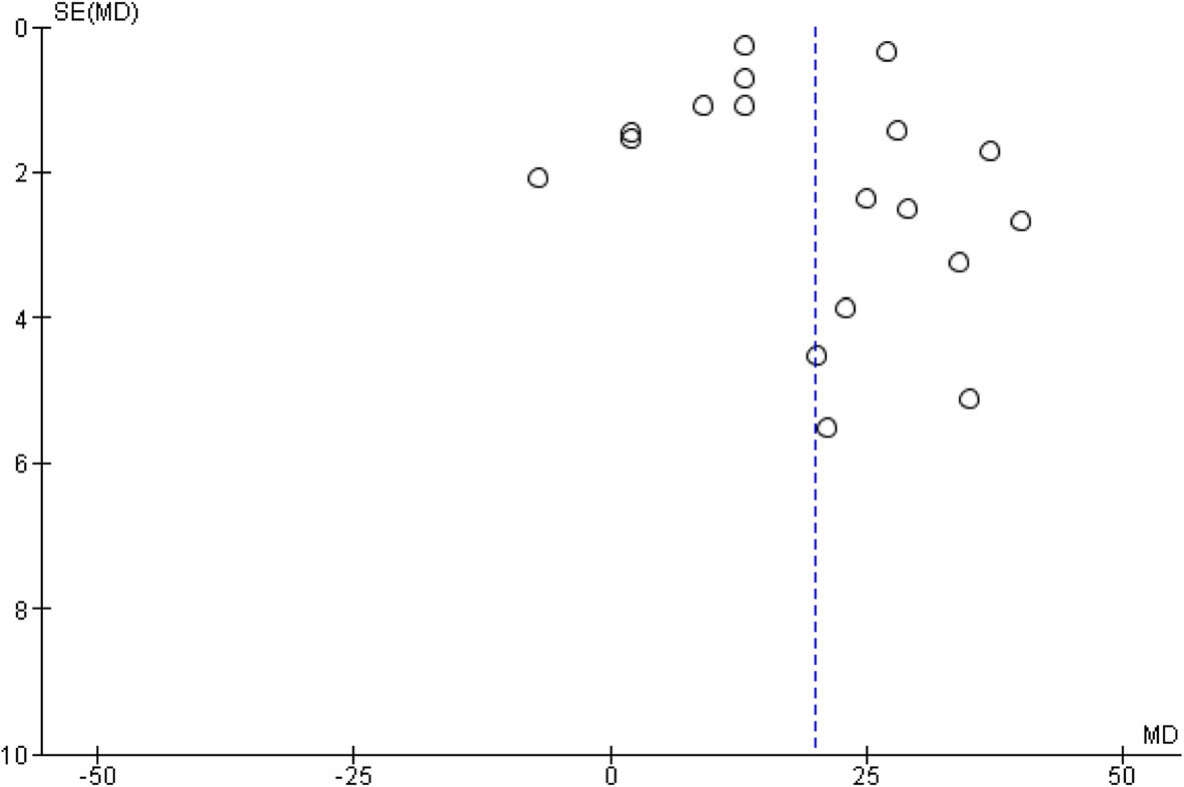
Publication biases**.** A funnel-plot illustrating publication biases for included studies comparing heart rate between postural orthostatic tachycardia syndrome versus healthy participants

Figure 5 illustrates a funnel-plot for publication biases among studies included in comparing HR between POTS versus healthy groups. Medium sample sized studies at the middle of the funnel-plot were more symmetrically distributed as compared to large sample sized studies at the top. This suggests heterogeneity of the study estimates as well as likely publication bias favoring studies with medium sample sizes than large sample sizes.

## Discussion

Age, sex, race, BMI, physical fitness and circadian rhythm are among a number of factors that have been reported to physiologically influence HRV. HRV in patients with POTS is no exception. This study was aimed to compare POTS patients versus healthy patients, in terms of their HR and HRV after HUTT, by systematic review and meta-analysis of available published literature.

From the results of our study, mean difference for TDM outcome measures between POTS versus healthy participants were found to be; RR interval = − 162.89 (− 197.93, − 84.07), *P*-value< 0.05; rMSSD = − 15.16 (− 18.28,12.03), *P*-value< 0.05. In this case, both outcomes showed statistically significant results that illustrate lower HRV in terms of TDM measure in the POTS group than in the comparison groups. Despite authors regarded R-R interval and rMSSD separately, it worth to note that rMSSD is calculated from R-R interval and they are directly proportional to one another. These findings concur with available base of literatures by; *De Wandele* et al.(2014) [42], *Galland* et al. (2008) [43], *Gergont* et al. (2019) [44], *Lewis* et al. (2013) [45] and *Pengo* et al.(2015) [46], ,all of whom reported reduced HRV in POTS than in non-POTS patients or otherwise heathy individuals. On the other hand, mean differences for FDM outcome between POTS versus healthy participants were: LF, = − 80.89 (− 211.37, 49.58), *P*-value> 0.05; HF, = − 113.20 (− 275.52, 49.13), *P*-value> 0.05 and HF (n.u) = − 0.03 (− 0.11, 0.04), all of which did not show statistically significant results that POTS patients have lower HRV than healthy participants in terms of FDM. Our study’s LF results align with those reported by *Mallien* et al. (2012) but contradict with those reported by *Stewart* et al. (2006). Our results for HF align with those reported by *Ocon* et al. (2009) but contradicts with those of *Freitas* et al. (2005).

Moreover, from our study, LF/HF- ratio was found to be 0.29 (− 0.25, 0.83), *P*-value> 0.05; LF (n.u), = 0.05 (− 0.04, 0.13), *P*-value> 0.05; all of which showed higher HRV in POTS patients in comparison to healthy participants in terms of FDM without reaching statistical significance. Our results for LF/HF-Ratio align with those reported by *Yoshida* et al. (2014) and contradict with those reported by *Mallien* et al. (2014). Our LF(n.u) results align with those previously reported by *Medow* et al. (2014) but contradict those reported by *Ocon* et al. (2009).

Regarding HR, our study strongly shows a statistically significant higher HR in POTS than healthy patients following HUTT with a mean difference of 19.88(15.24, 24.52), *P*-value< 0.05. These results align with the majority of previously published literature but contradict with those reported by *Meier* et al. (2006) who reported otherwise.

Authors of this study believe that the reasons for variations and contradictions among all aforementioned studies and their outcomes to greatly be due to methodological reasons, especially inadequate and/or improper matching of participants. Authors, therefore, recommend more robust researches to be conducted in the topic, matching participants with age, gender, ethnicity, BMI, physical fitness and circadian rhythm.

Amid a number of theories explaining low HRV in POTS patients, one is a hyperadrenergic state [15, 36, 47]. Physiologically, POTS patients have been reported to have increased sympathetic activity following a suggested hyperadrenergic state. Another theory for low HRV in POTS patient is, distal denervation predominantly in lower extremity, with preserved cardiac innervation leading to lower extremity anhidrosis, impaired norepinephrine spillover in the lower extremities and decreased muscle sympathetic activity recruitment in the lower extremity in response to a nitroprusside-induced hypotensive stimulus [38, 48, 49]. Other studies have reported hypovolemia, decreased venous posture in an upright position, baroreflex abnormality and cardiac deconditioning to contribute [50].

Despite promising results, the results of this study need to be addressed with care. This follows a number of bias sources that were encountered and assumptions that were made during the conduction of this study. Different studies involved different number sample sizes and none of these twenty studies reported to have calculated the required sample size prior to their conduction. Furthermore, improper matching as explained earlier, different methods of inducing orthostasis including variable angles of tilt from 40^0^ to upright; different methods of measuring outcomes including the use of ECG and/or Holter device and different durations for measurement of HRV parameters and HR (i.e. short term or 24 h term). Moreover, at the review level, a sensitivity analysis was conducted due to high heterogeneity observed across different parameter outcomes especially in the FDM and HR. Three peculiar studies were eliminated but none of the initially calculated results changed their statistical significance. Again, rMSSD has been shown to have an association with HR which could have confounded our results [51]. From fewer otherwise eligible studies reporting the two parameters, meta-regression could not be conducted. To help mitigate biases, authors firstly appraised all eligible studies and used team work in conducting database search and data extraction. To mitigate reporting biases, PRISMA tools were used in the study writeup.

## Conclusion

Despite a number of unavoidable sources of biases, it worth to note that despite the massively supported fact that POTS patients have a higher HR than healthy patients after HUTT, POTS patients have lower HRV in terms of TDM but not in terms of FDM. It follows that HR and TDM analyses of HRV are more reliable than FDM analysis in differentiating POTS patients from a health participant. We, though, call upon more extensive observational (preferably sensitivity and specificity studies) and interventional studies to further mitigate biases encountered in this study.

## Data Availability

The datasets used and analyzed during the current study are available from the corresponding author on reasonable request.

## Abbreviations

BMI: Basal metabolic index
ECG: Electrocardiogram
FDM: Frequency domain measure
GM: Gui Ming (author)
HF: High frequency power
HF(n.u): High frequency normalized unit
HR: Heart rate
HRV: Heart rate variability
HUTT: Head-up tilt test
JS: Joel Swai (author)
LF: Low frequency power
LF(n.u): Low frequency normalized unit
MeSH: Medical subject headings
OH: Orthostatic hypotension
OI: Orthostatic intolerance
POTS: Postural orthostatic tachycardia syndrome
PRISMA: Preferred reporting items for systematic reviews and meta-analyses
rMSSD: Square root of the mean of squares of successive R-R waves
RR-Interval: Interval between R and R waves of the electrocardiogram
TDM: Time domain measure
TR: Tibera Rugambwa (author)
XZ: Xiexiong Zhao (author)
ZH: Zixuan Hu (author)
